# The Effects of Secretory IgA in the Mucosal Immune System

**DOI:** 10.1155/2020/2032057

**Published:** 2020-01-03

**Authors:** Yue Li, Liang Jin, Tongxin Chen

**Affiliations:** ^1^Department of Rheumatology/Immunology, Children's National Medical Center, Shanghai Children's Medical Center, Shanghai Jiao Tong University School of Medicine, Shanghai, China; ^2^Division of Immunology, Institute of Pediatric Translational Medicine, Children's National Medical Center, Shanghai Jiao Tong University School of Medicine, Shanghai, China; ^3^Med-X Research Institute, School of Biomedical Engineering, Shanghai Jiao Tong University, Shanghai, China

## Abstract

Immunoglobulin A (IgA) is the most abundant antibody isotype in the mucosal immune system. Structurally, IgA in the mucosal surface is a polymeric structure, while serum IgA is monomeric. Secretory IgA (sIgA) is one of the polymeric IgAs composed of dimeric IgA, J chain, and secretory component (SC). Most of sIgAs were generated by gut and have effects in situ. Besides the function of “immune exclusion,” a nonspecific immune role, recent studies found it also played an important role in the specific immunity and immunoregulation. Thanks to the critical role of sIgA during the mucosal immune system homeostasis between commensal microorganisms and pathogens; it has been an important field exploring the relationship between sIgA and commensal microorganisms.

## 1. Introduction

Mucosal surfaces provide a physical barrier to defend foreign pathogens as well as to involve the tolerance of the commensal microbes or harmless food antigens. The protection of these surfaces is ensured by the mucosal immune system, designated as the mucosa-associated lymphoid tissues (MALT), which consists of mucus layers and epithelium cells, together with lymphoid tissues and immune molecules in the mucosal lamina propria [1, 2]. The immunoglobulin A (IgA) is the predominant antibody isotype in the mucosal immune system, which widely exists in the gastrointestinal tract, respiratory tract, vaginal tract, tears, saliva, and colostrum. Normally, serum IgA shows a monomeric structure, while the mucosal IgA shows polymeric. The function of the former is still unclear [3]. Distinctively, we designated the subtype of IgA composed of two monomeric IgA, secretory component (SC), and J chain as secretory IgA (sIgA) [4], which is the major effective form of mucosal IgA. There are also trimeric sIgA, tetrameric sIgA, and larger polymeric IgA in the upper respiratory tract of healthy humans. Among them, tetrameric IgA has a broad neutralizing function against influenza viruses [5]. Previous studies showed that mucosal immunity is segregated from systemic immune responses [6, 7]. The mucosal system can maintain the balance in the mucosal immunity between the commensal microorganisms and defenses the pathogens on the mucosal surface because of sIgA contribution [8]. Conversely, research showed there was a lack of IgA-secreting B cells in neonates until exposure to bacteria, suggesting that the commensal microorganisms were able to induce sIgA secretion [9, 10]. In humans, sIgA was also a major immunoglobulin in colostrum, which integrates the mucosal immune systems of mother and child for great protective functions [11]. However, selective IgA deficiency, a common primary immunodeficiency, often presents an asymptomatic phenotype or mild consequences, which may question the significance of IgA [12]. In this review, we will discuss the mechanism of sIgA generation and their function during the mucosal immune response.

## 2. Structure of sIgA

As an immunoglobulin, IgA has two identical heavy chains and two identical light chains. There is a flexible hinge region to separate above chains into two Fab regions-binding the antigens and an Fc region-mediating the effects [13]. In human, IgA has two subsets termed IgA1 And IgA2. The hinge region of IgA1 contains a 13-amino acid longer extension, ranging from three to six, variable O-glycan substitutions but not in IgA2 [4, 14]. Although both IgA1 and IgA2 carry N-linked glycosylation sites at every heavy chain, the latter has two additional N-linked oligosaccharides that may resist to the proteolytic activity of the bacteria in secretions better than the former [12].

Dimeric IgA (dIgA) was made of two monomeric IgAs linked in the penultimate Cys residues of their Fc regions via J (joining) chain and IgA2 is preferred. J chain is a small polypeptide to form pentameric IgM and dimeric IgA, but little is known about the function of J chain due to the technical limitation [15]. When one dIgA is bound to the polymeric immunoglobulin receptor (pIgR) at the basolateral side of the epithelium thereby transported to the luminal side, the dIgA-binding portion of the pIgR is cleaved to form the molecule sIgA [16]. The pIgR fragment of sIgA is called secretory component (SC) to support the stability of sIgA [17].

Although both IgA1 and IgA2 can form sIgA, the variety of subclass proportions will happen in different tissues. For example, there are 80 to 90% IgA1 in nasal and male genital secretions, 60% IgA1 in saliva, and 60% IgA2 in colonic and female genital secretions [4].

## 3. Induction of sIgA

The mucosal immune system can principally be divided into inductive sites and effector sites [18]. The classical sIgA inductive sites are gut‐associated lymphoid tissue (GALT) including Peyer's patches (PPs), isolated lymphoid follicles (ILFs), and mesenteric lymph nodes (MLNs). The GALT contains at least 80% plasma cells (PCs) and 90% sIgA of the body [19]. It has been estimated that approximately 3 g of sIgA is exported into the gut lumen of an adult human every day [20]. Craig and Cebra reported that Peyer's patches (PPs) were the principal precursor source of IgA^+^ PCs [21]. In addition, the nasopharynx-associated lymphoid tissues (NALT) and the bronchus-associated lymphoid tissues (BALT) are also mucosal immune inductive sites [22].

### 3.1. Antigen Presentation of DCs

Peyer's patches were covered with an epithelial monolayer, follicle-associated epithelium (FAE), containing microfold cells (M cells) inside. Beneath the FAE, subepithelial dome (SED) covers the B follicles, while the DCs exists in the SED [23]. Mucosal antigens were captured by the underlying DCs by extending their dendrites [24] or through the transcytosis of M cells [25]. Evidence showed both FAE and small intestine goblet cells (GCs) were involved in the antigen uptake [26, 27].

Upon antigen presentation by DCs, T cells and B cells were activated and IgA class switch recombination (CSR) were mediated in the mucosal B cells, which replaced the immunoglobulin heavy chain C regions (C*μ*) with the downstream C*α* gene [28]. There are long repetitive switch (S) regions preceding C*μ* and downstream C*α*. Activation-induced cytidine deaminase (AID) converts cytosines in S regions to uracils by the deamination lesions, which instigates the CSR. These uracils lesions are subsequently removed by two DNA repair factors, resulting in DNA double-strand breaks (DSBs) and recombination between upstream S region and downstream S region [29]. Additional, TGF-*β* has a critical role in this process, which binds to the TGF*β*R on the surface of B cells, thereby leading to the SMAD3/4 and Runx3 activation and subsequently combining with the TGF-*β* responsive elements in the I*α* promoter of the IgA heavy chain gene [30].

### 3.2. T-Dependent (TD) Mechanism and T-Independent (TI) Mechanism

In terms of the participation of T cells in this process, the IgA CSR were divided into T-dependent (TD) mechanism and T-independent (TI) mechanism. The former required interaction between CD40 on the surface of B cells and its ligand CD40L derived from T cells, resulting in high-affinity antigen-specific IgA production to neutralize the pathogens [31]. T follicular helper (Tfh), Foxp3 ^+^Treg, and Th17 cells are involved in promoting the IgA response in the intestine by the release of various cytokines, such as IL‐4, IL‐5, IL‐6, IL‐10, IL‐13, IL-17A, and IL-21, to further promote the CSR to IgA [32]. However, evidences have demonstrated that CD40 deficiency in human and mice retain IgA production [33, 34], suggesting that CSR to IgA could occur via TI mechanism, which produced commensal-reactive IgA through innate immune cells such as innate lymphoid cells (ILCs) and plasmacytoid dendritic cells (pDC) [2, 31, 35]. During the TI pathway, BAFF (B-cell activating factor of the TNF family) and APRIL (A proliferation-inducing ligand), two members of TNF family, are responsible for stimulating CSR to IgG or IgA in human [36].

### 3.3. sIgA Effector Sites

IgA gene sequences of wild type mice present substantial somatic hypermutation (SHM), which reveal the germinal center (GC) origin in the PPs [37]. Due to the food antigens and the microbiota in the gut, GC in the PPs constantly presents and B cells can repeatedly enter into preformed GC in the recirculation, which contributes to the B cell affinity maturation and the formation of long-lived plasma and memory B cells [38]. After terminal differentiation to plasmablasts and plasma cells (PCs), IgA^+^ B cells migrate into the bloodstream and prefer to home the mucosal inductive sites preferentially and other secretory effector sites [18]. Migration and interaction happened between different sIgA inductive sites and sIgA effector sites. Lung dendritic cells can also induce IgA CSR and generate protective gastrointestinal immune responses [39]. Lactating mammary glands are also vital sIgA effector sites, and the antigenic stimulation from maternal gut and airways could result in the sIgA specificity for intestinal and respiratory pathogens [40].

## 4. Functions of sIgA

As a primary antibody class found in various external secretions, sIgA has unique structural and functional features not observed in other antibody classes. Classically, sIgA eliminates the pathogens with immune exclusion via nonspecific immunity [8]. Apart from that, sIgA plays an indispensable role in specific immunity elicited by pathogens. For example, sIgA can be elicited by mucosal vaccines against influenza virus and colitogenic bacteria in inflammatory bowel disease (IBD) [41, 42]. One of the hallmark characters in the mucosal immune system is the microbe colonization [43]. A study has confirmed that both TI and TD immune responses are involved in coating different commensal bacteria with sIgA [44]. In conclusion, response to the pathogens and induction of tolerance under normal conditions such as innocuous food antigens or commensal bacteria are dual functions of sIgA to maintain the homeostasis in mucosal sites.

### 4.1. Immune Exclusion

Traditionally, IgA is thought as a noninflammatory antibody at mucosal sites. Due to its polymeric structure and the oligosaccharide side chains of SC [45], sIgA is concentrated in the mucus out layer [46], noncovalently cross-linking microorganisms, promoting microorganisms clump together in situ. Furthermore, the abundant hydrophilic amino acids of IgA Fc and glycosylation of IgA and SC result in the hydrophily of sIgA, to entrap microorganisms [4], and then peristaltic bowel movements help remove the bacteria clumps. The process of agglutination, entrapment, and clearance processes are called immune exclusion [47].

### 4.2. Multiple Neutralizing Properties

Immune exclusion presents the nonspecific immunity function of sIgA. sIgAs have more extensive protective functions. Firstly, sIgA coating and the steric hindrance help block microbial adhesins to interact with the epithelium, sIgA can also inhibit specifically pathogens by direct recognition of receptor-binding domains such as reovirus type 1 Lang (T1L) [48]. The advanced glycosylated IgA heavy chain and SC serve as competitive inhibitors of the pathogen adhesion process [47]. Blocking pathogens from interacting with epithelial cells is not the exclusive mechanism by which sIgA exerts its protective function. In addition, sIgA may have direct effects on impacting the bacterial viability or changing pathogenicity. For example, sIgA can interact with flagella to inhibit the *Salmonella* bacterial motility [49], as well as protect from cholera toxin-induced fluid accumulation in a ligated intestinal loop model [36]. SC is proved to interact with a surface protein of *Streptococcus pneumoniae*, choline binding protein A (CbpA) [8]. And the galactose residues of free SC could also neutralize *Clostridium difficile* toxin A and enteropathogenic *E. coli* intimin [50].

### 4.3. sIgA and Receptor

Fc*α*RI is the most important IgA host receptor, widely expressed in cell types including neutrophils, eosinophils, monocytes, and macrophages. [51], to mediate biological effects such as antibody-dependent cellular cytotoxicity (ADCC), phagocytosis, antigen presentation and release of cytokines, superoxide generation, calcium mobilization, and degranulation [52]. Because of the similar IgA binding site for Fc*α*RI and pIgR, sIgA-Fc*α*RI binding is partly hampered by the steric hindrance of SC. Although sIgA is not able to activate phagocytosis by neutrophils or Kupffer cells, sIgA can initiate respiratory burst activity by neutrophils [53]. This process is dependent on the expression of Mac‐1 (CD11b/CD18), suggesting that sIgA needs this integrin co-receptor to bind or activate Fc*α*RI [54]. Besides Fc*α*RI, sIgA has also been described to interact with pIgR, transferrin receptor (Tfr/CD71), asialoglycoprotein receptor (ASGPR), Fc*α*/*μ*R, FcRL4, and dendritic cell-specific intercellular adhesion molecule-3-grabbing non-integrin (DC-SIGN) [55]. SIgA immune complexes can reverse transport back into the lamina propria via the Tfr on epithelial cells [56] or via interaction with dectin-1 through microfold cells (M cells) [57]. Besides, sIgA immune complexes of the lamina propria were recognized by DC-SIGN and taken up by subepithelial DCs [58]. The SIGNR1, the mouse homolog of DC-SIGN, can also interact with sIgA and induce the tolerogenic DCs. The sIgA-DCs generate the expression of regulatory T cell, which indicates the potential immunoregulation function of sIgA [59]. Furthermore, polymeric sIgA of the lamina propria also binds and excretes antigens back to the lumen using polymeric Ig receptor-mediated transcytosis across the epithelial cells [60].

### 4.4. sIgA and Commensal Organisms

As is well-known, some patients with selective IgA deficiency show clinically asymptomatic or mild infections but have a higher risk of allergy and autoimmunity [61]. Researches show that both secretory IgM and systemic IgG can replace part of sIgA and establish the second defense line [62, 63]. Is sIgA a redundant component in the immune system? Emphasizing the effects of sIgA on the commensal microorganisms may explain its significance. The modification of microbiota is one of the most features in the selective IgA deficiency patients, and pIgR deficiency mice could inhibit different microbiota [62, 64]. As mentioned above, TD and TI mechanisms help with different-affinity sIgA. Low-affinity antibodies are specific for diverse commensal microorganisms, inclining to host-commensal mutualism, while high-affinity antibodies defend the pathogens [31]. In addition, the TD and TI mechanisms mediate the different sIgA coatings with bacteria [44], leading to different recognition by epithelial cells and DCs [65], and the level of sIgA coating varies between different members of the microbiota [10]. In neonates, maternal IgA is the sole source of sIgA. Evidence revealed that mice which did not receive sIgA in breast milk had a significantly distinct gut microbiota, and these differences were persistent and magnified in adulthood [66]. In addition, the maternal IgG and IgA were reported to inhibit the mucosal T helper cell responses, which revealed the TI mechanisms maintain the host-commensal mutualism in early life [67]. In the programmed cell death 1- (PD-1-) deficient mice, sIgAs were dysregulated and led to the change of microbial communities [68]. Therefore, we propose that TD and TI mechanisms might have synergetic roles in microorganism diversity and commensal homeostasis. However, the precise mechanisms of regulation on the maintain the homeostasis and the memory B cells diversity and long-lived gut plasma cells are ready to clear [69].

Of note, the relationship between intestinal microbes and IgA is bilateral. The microbiota also modulates the sIgA distribution. A classic example is segmented filamentous bacterium (SFB), a commensal bacterium, remarkably inducing and stimulating multiple types of intestinal lymphoid tissues that generate sIgA [70]. Furthermore, Proietti et al. also proved the microbiota can release ATP to limit the generation of sIgA [71]. However, the effects of the microbe on the distribution of high-affinity and low-affinity sIgA are uncertain. Pabst suggested the model that all microorganisms could induce the high-affinity antibodies albeit vary different immunostimulatory activities and kinetics [31].

## 5. Conclusion

The mucosal system is the first line of immune defense while the sIgA is the first line of mucosal immunity. In this review, we have described the significant dual function of sIgA for maintaining immune homeostasis in mucosal compartments and the complexity of the sIgA action modes. sIgA presents a great latent capacity in shaping both the infant mucosal immunity and commensal microbial environments. Since breast milk is the main source of sIgA as well as a fundamental immune component for neonates, it offers a potential therapy in the clinics [66].
